# New Expensive Anticancer Agents: Which Role Is Played by Quality-Adjusted Life-Years in the Selection of a Specific Treatment?

**DOI:** 10.1200/JGO.2016.006361

**Published:** 2016-08-10

**Authors:** Andrea Messori

**Affiliations:** ESTAR, Regional Health Service, Firenze, Italy

## To the Editor:

Koczwara et al^1^ emphasize that dissemination and implementation science should broaden its reach beyond its current geographic limits (including the United States, the United Kingdom, Canada, and Australia). However, even within these limits, critical issues remain in the field of oncology. In fact, although evidence behind cancer control strategies may not differ around the globe, other critical points, such as how pharmacoeconomic concepts are applied in various health care systems, show important geographic variations that deserve close scrutiny.

In 2015, a task force of oncologists coordinated by ASCO developed a complex consensus document entitled “A Conceptual Framework to Assess the Value of Cancer Treatment Options,”^2,3^ which examined the main determinants of effectiveness for current anticancer treatments along with cost. In particular, the data on effectiveness and cost were combined to produce a series of scores that patients, together with their oncologists, can use to select their treatment among the available options.

In the same year, another project with similar characteristics was carried out by the European Society for Medical Oncology (ESMO).^4^ The ESMO project aimed to determine the magnitude of clinical benefits from anticancer therapies by using a standardized approach. The report showed an important difference because costs were not included in the analysis.

In 2016, the International Society for Pharmacoeconomics and Outcomes Research (ISPOR) published a series of comments on the ASCO project.^5^ The most relevant observation was that the ASCO task force did not embrace in its conceptual framework the use of quality-adjusted life-years (QALYs). In contrast, according to the ISPOR experts, “the health economics and outcomes research field is substantially invested in using both QALYs and cost-effectiveness analyses as tools to support the difficult health care resource allocation decisions that societies face.”^5(p1)^ In practice, these experts disagreed on the task force’s decision to avoid the use of QALYs.

The main purpose of QALYs is to quantify the magnitude of clinical benefit within an analysis that examines effectiveness and cost. Hence, the question of whether to use QALYs remains outside the field of interest of the ESMO tool but is pertinent to the application of the ASCO tool.

The main reason given for developing the ASCO tool was that patients often are confronted with enormous treatment expenses, so “the goal of describing a relationship between the cost of an agent or regimen and the clinical benefits it delivers takes on great importance.”^3(p1)^ Furthermore, the oncologist plays “an important role in providing a comparative assessment of the various treatment options available,” including “information about the clinical impact expected from the different options and their relative financial implications.”^3(p1)^ This objective (ie, a shared decision by patient and oncologist on the treatment) was identified in the context of the US setting, where in many cases, a large proportion of the anticancer drug cost is directly incurred by the patient.^6^ Given that the ASCO tool is aimed at facilitating patient decisions, it is understandable that all technical parameters in this tool (eg, the QALY) were avoided and that the information on costs and effectiveness was based on scores that do not belong to the field of specialized research.

The ISPOR perspective on the ASCO document is different. ISPOR is a society of health economists and outcomes researchers and, according to its mission, does not allow patients to become members. Use of the QALY, therefore, is in line with the nature of a specialized scientific society, so if the people to whom these evaluation tools are directed already know the background behind the QALY concept, the use of this parameter in a practical document increases the effectiveness of communication. Although not all the authors of the ISPOR document were European, and many were from the United States, the ISPOR comments essentially reflect a European perspective in which national health systems are responsible for reimbursing the new anticancer agents and patients are requested to contribute little (or no) out-of-pocket money.

In conclusion, the degree of technicality of a document that guides the selection of anticancer treatments must be different depending on whether the patients are the intended audience of the document itself. In this context, the geographic setting and particularly the characteristics of the local health care system are extremely important.
